# Metabolomics in Traditional Chinese Medicine for Diabetes Mellitus: Mechanistic Insights, Biomarker Discovery, and Clinical Application Prospects

**DOI:** 10.1155/ianc/8261773

**Published:** 2026-05-15

**Authors:** Junhui Song, Gen Lin Li, Suhui Wu, Hanbing Li

**Affiliations:** ^1^ School of Medicine, Henan University of Chinese Medicine, Zhengzhou, Henan, China, hactcm.edu.cn; ^2^ Henan Province Health Aging Industry Research Center, Henan University of Chinese Medicine, Zhengzhou, Henan, China, hactcm.edu.cn

**Keywords:** analytical workflow, biomarker identification, diabetes mellitus, metabolite profiling, metabolomics, traditional Chinese medicine

## Abstract

**Objective:**

This review provides a comprehensive summary of recent advancements in metabolomic analysis within traditional Chinese medicine (TCM) for the treatment of diabetes mellitus. It focuses on the standardized profiling of syndrome‐specific metabolites, the identification of bioactive compounds using high‐throughput techniques, and the elucidation of mechanisms based on metabolic pathways. The review aims to establish a reproducible analytical framework for TCM metabolomics, which includes identifying active compounds, clarifying mechanisms of action, and assessing therapeutic efficacy. By synthesizing the current body of evidence, this work seeks to provide a scientific foundation for future research, enhance the integration of metabolomics with TCM theory, and support the modernization and global acceptance of TCM in diabetes care. Ultimately, it addresses key challenges, such as the subjective nature of syndrome diagnosis and the complexity of multicomponent interactions.

**Subjects and Methods:**

A systematic review of the peer‐reviewed literature was conducted using PubMed, Web of Science, CNKI, and Wanfang Data for studies published up to 2025. The review included original research, reviews, and clinical trials that utilized metabolomic techniques (LC‐MS/MS, GC‐MS, and 600 MHz NMR) and standardized workflows (sample preparation, derivatization, instrument analysis, and data processing) in diabetic models. The qualitative synthesis focused on high‐throughput analytics, multivariate statistics (principal component analysis/partial least squares‐discriminant analysis [PCA/PLS‐DA] with 7‐fold cross‐validation and CV‐ANOVA, *p* < 0.05), and metabolite identification via the human metabolome database (HMDB)/METLIN metabolite database (mass error < 5 ppm); furthermore, these data were integrated with network pharmacology or multiomics approaches.

**Results:**

Metabolomics has revealed distinct metabolic profiles for TCM syndromes. Specifically, the Qi‐Yin deficiency (QYD) syndrome is associated with altered levels of L‐glutamate (variable importance in projection [VIP] > 1, *p* < 0.05) and arachidonic acid (limit of detection [LOD] = 0.01–0.1 ng/mL), as well as the accumulation of turbid toxins involving pantothenate and CoA biosynthesis. Key hypoglycemic bioactive compounds, such as epicatechin and berberine, were identified using ultraperformance liquid chromatography‐quadrupole time‐of‐flight mass spectrometry (UPLC‐QTOF‐MS), with a mass error of < 5 ppm. Mechanistic studies have shown that TCM works through multiple pathways, including improving insulin resistance (e.g., mulberry leaves modulating amino acid/lipid metabolism); protecting β‐cells (e.g., timosaponin BII restoring phosphatidylserine levels); regulating glycolipid metabolism (e.g., Huanglian decoction influencing energy‐related metabolites); modulating gut microbiota (e.g., Gegen Qinlian decoction altering bile acids and short‐chain fatty acids); and preventing complications (e.g., *Sophora flavescens* affecting oxidative stress through glycerophospholipid metabolism). Furthermore, metabolomics enabled efficacy comparisons, highlighting improved outcomes with nanoformulations or exercise‐TCM combinations.

**Conclusions:**

Metabolomics provides a powerful approach to objectively assess TCM syndromes, clarify multitarget mechanisms, and comprehensively evaluate efficacy in diabetes treatment, which aligns well with TCM’s holistic principles. Despite existing challenges, such as the lack of standardized TCM syndrome classification and the complexity of metabolomic data (e.g., overlapping metabolite signals and multipathway crosstalk), future studies should focus on rigorous experimental designs, standardized protocols, and multiomics integration to promote biomarker discovery, personalized TCM, and its global integration into diabetes management.

## 1. Introduction

Diabetes mellitus is a chronic condition characterized by hyperglycemia, which results from either absolute or relative insulin deficiency and impaired insulin utilization. The prevalence of diabetes has been steadily increasing in recent years, becoming a significant global public health issue. According to the 2021 report from the International Diabetes Federation (IDF), approximately 537 million adults (aged 20–79 years) worldwide are affected by diabetes, with China having the highest number of adult diabetes patients. Type 2 diabetes mellitus (T2DM) accounts for over 90% of these cases [1]. Diabetes presents substantial health risks, often accompanied by multiple complications that severely affect patients’ quality of life and overall health. While current therapeutic strategies can effectively control blood glucose levels, they are limited in their ability to fully replicate physiological insulin secretion patterns and cannot completely halt the progression of the disease.

The development of metabolomics began in the mid‐1990s as an emerging analytical field focused on the qualitative and quantitative analysis of small‐molecule metabolites (< 1 kDa) in biological systems. In traditional Chinese medicine (TCM) research, metabolomics addresses unique analytical challenges, such as complex matrices with coexisting primary and secondary metabolites, low‐abundance bioactive compounds, and inconsistent sample processing. These challenges represent critical barriers to the modernization of TCM. However, metabolomics provides a holistic and quantitative approach to analyzing metabolite sets in biological systems, offering functional insights into physiological, pathological, and pharmacological processes. After decades of advancement, metabolomics has found broad applications in TCM research. TCM, with its multicomponent, multitarget, and multipathway properties, offers unique advantages in diabetes treatment by regulating systemic metabolic networks. This aligns closely with metabolomics’ ability to capture simultaneous changes across multiple metabolic pathways. This review summarizes recent progress in the application of metabolomics to TCM treatment of diabetes, aiming to provide a comprehensive theoretical foundation for its use in this field.

## 2. Overview and Principles of Metabolomics

Metabolomics, as a key component of systems biology, aims to systematically analyze all small‐molecule metabolites such as carbohydrates, lipids, amino acids, and organic acids within an organism to reveal its physiological and pathological states and responses to external stimuli. Metabolomics is an emerging discipline that studies small‐molecule metabolites in biological systems, following genomics, transcriptomics, and proteomics [2]. Since its introduction by Nicholson’s team at Imperial College London, it has been widely applied in biomarker discovery, disease mechanisms, drug target identification, and translational research related to drug responses [3, 4].

Metabolomics research relies on standardized high‐throughput analytical techniques: (1) LC‐MS/MS (Thermo Q Exactive): C18 column (2.1 × 100 mm, 1.9 μm); mobile phase (A: 0.1%formic acid; B: acetonitrile); gradient elution (0–5 min: 5%–30%B; 15–20 min: 80%–95%B); and ESI + mode, m/z 50–1000; (2) GC‐MS (Agilent 7890A–5975C): DB‐5MS column and BSTFA + 1%TMCS derivatization (70°C, 30 min); and (3) NMR (Bruker AVANCE III 600 MHz): NOESY‐presat pulse sequence, TSP reference (*δ* = 0.00), 128 scans. These methods effectively separate and detect metabolites in complex biological samples. For instance, LC‐MS has become a mainstream technique in metabolomics due to its high sensitivity and broad applicability [5, 6]. A summary of the high‐throughput analytical techniques commonly utilized in metabolomics research is presented in Table 1. The abbreviations used in Table 1 are defined as follows: lysophosphatidylcholine (LPC), sphingomyelin (SM), phosphatidylethanolamine (PE), lysophosphatidylethanolamine (LPE), triglyceride (TG), and diglyceride (DG).

**TABLE 1:** High‐throughput analytical techniques commonly used in metabolomics research.

Analytical technique	Instrument model	Key experimental parameters	Remarks
LC‐MS/MS	Thermo Q Exactive	Column: C18 (2.1 × 100 mm, 1.9 μm); mobile phase: A (0.1% formic acid), B (acetonitrile); gradient elution: 0–5 min (5%–30% B), 15–20 min (80%–95% B); ionization mode: ESI+; m/z range: 50–1000	Mainstream technique with high sensitivity and broad applicability
GC‐MS	Agilent 7890A‐5975C	Column: DB‐5MS; derivatization: BSTFA + 1% TMCS (70°C, 30 min)	Suitable for the detection of volatile and semivolatile metabolites
NMR	Bruker AVANCE III 600 MHz	Pulse sequence: NOESY‐presat; reference standard: TSP (*δ* = 0.00); number of scans: 128	Nondestructive detection, suitable for qualitative and quantitative analysis of metabolites

While these analytical platforms and workflows lay the foundation for metabolomics research, the rigor and reproducibility of results heavily depend on robust quality control (QC) strategies, batch effect correction, and cross‐cohort validation—key aspects that require more critical evaluation in diabetes metabolomics studies. To address this, recent guidelines and studies have delineated clear best practices: the QC omics consortium [7] proposed a comprehensive, implementable QC protocol that systematically addresses batch effects, normalization strategies, internal standard selection, and outlier detection, ensuring data accuracy and reproducibility across experiments and laboratories. This protocol specifically emphasizes the importance of QC sample inclusion, background noise correction, and missing data handling—critical steps for avoiding misleading interpretations in diabetes metabolomics, where subtle metabolic shifts are closely linked to disease progression.

**FIGURE 1:**
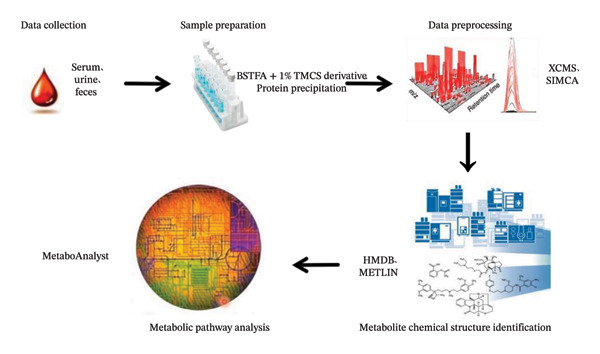
Schematic workflow of metabolomics analysis.

**FIGURE 2:**
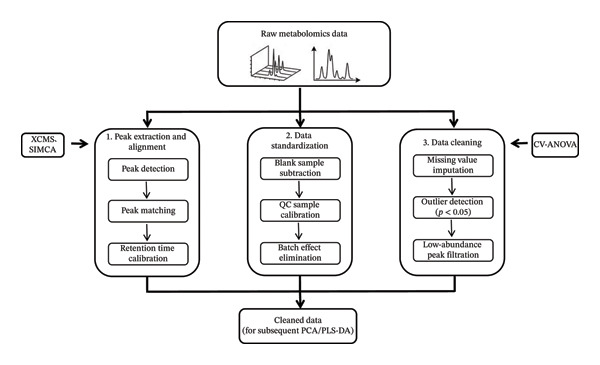
Schematic diagram of common types and workflows of metabolomic data pre‐processing.

Figure 1 depicts the standardized and classical analytical workflow of metabolomics, which consists of five core sequential steps. For data collection, the primary sample types include serum, plasma, urine, feces, and liver tissue. Sample preparation involves targeted preprocessing strategies, such as derivatization with BSTFA containing 1% TMCS (70°C for 30 min) for GC‐MS analysis and protein precipitation for LC‐MS/MS analysis, aiming to improve the detectability of metabolites in complex biological matrices. Data preprocessing is a critical step to ensure data reliability: raw data are processed via XCMS and SIMCA software, combined with rigorous QC strategies including QC sample insertion, batch effect correction, and seven‐fold cross‐validation. Retention time calibration is performed to eliminate the deviations in retention time and m/z values of metabolites across different samples, thus ensuring the accuracy of peak matching. Figure 2 depicts the complete procedure for data preprocessing. The chemical structure identification of metabolites is based on the HMDB and METLIN metabolite database, with a stringent mass error threshold of < 5 ppm. Potential specific biomarkers are screened using the criteria of VIP > 1 combined with *p* < 0.05. Further metabolic pathway analysis of the identified differential metabolites is conducted by integrating bioinformatic tools. This standardized metabolomic workflow provides a reproducible analytical framework for metabolomic research and serves as a technical basis for elucidating the material basis and therapeutic mechanisms of diabetes treatment.

In data analysis, quantitative metabolite profiling combined with multivariate statistics (PCA/PLS‐DA) facilitates the extraction of meaningful information from complex metabolic data, and results interpretation requires integration with biological context to ensure scientific validity [8, 9]. Furthermore, interpreting metabolomics results requires integration with biological context to ensure scientific validity and reliability. Combined with clinical data, metabolomics not only supports early diabetes diagnosis but also informs personalized treatment strategies [1, 10].

## 3. Applications of Metabolomics in TCM Research for Diabetes Treatment

### 3.1. Current Status of TCM in Diabetes Treatment

TCM’s understanding of diabetes is rooted in its unique theoretical framework, emphasizing “yin‐yang imbalance” (a core TCM concept referring to the disharmony of opposing yet complementary forces in the body) and “visceral dysfunction” (dysregulation of organs like the spleen, kidney, and lung) as fundamental causes. In TCM, diabetes is classified under “xiao ke” (wasting‐thirst syndrome), with core pathogenesis involving yin deficiency and dryness‐heat, primarily affecting the lungs, stomach, and kidneys, while involving the liver and spleen. Etiological factors include constitutional deficiency, improper diet, emotional disturbances, and irregular lifestyle. Main symptoms include polydipsia, polyuria, and emaciation. TCM theory posits that diabetes arises from dysfunctions in organs such as the spleen, kidneys, and lungs, with spleen deficiency and kidney yin deficiency as primary causes [11]. TCM emphasizes treatments that harmonize yin and yang, tonify the spleen and kidneys, and clear heat and moisten dryness to restore bodily balance. In clinical practice, the efficacy of TCM in diabetes treatment is widely recognized (Table 2). Some classic formulas and traditional herbal compounds have demonstrated potential mechanisms; for example, Gegen Qinlian decoction significantly reduced blood glucose and serum insulin levels in T2DM mice. Network pharmacology and bioinformatics analyses revealed that it regulates 82 corresponding proteins and 59 diabetes‐related biological pathways [12]. Jiangtang Sanhuang tablet, derived from Taohong Chengqi decoction from the Treatise on Cold Damage with modifications, alleviates T2DM by modulating gut microbiota and bile acid metabolism interactions [13]. Danggui Liuhuang decoction has been shown to reduce proinflammatory cytokines and vascular endothelial growth factor release by inhibiting the JAK2/STAT3 signaling pathway, thereby ameliorating endothelial dysfunction in T2DM [14].

**TABLE 2:** Commonly used Chinese medicines for diabetes: A summary.

Single herbs	TCM herb pairs	Chinese herbal compound formulas
*Astragalus membranaceus, Coptis chinensis, Rehmannia glutinosa, Panax ginseng, Panax quinquefolius, Dioscorea opposita, Ophiopogon japonicus, Pueraria lobata, Trichosanthes kirilowii, Anemarrhena asphodeloides, Salvia miltiorrhiza, Lycium barbarum, Scrophularia ningpoensis, Atractylodes lancea, Poria cocos, Alisma orientale, Zea mays*	*Astragalus membranaceus*–*Coptis chinensis*, *Astragalus membranaceus*–*Ophiopogon japonicus*, *Astragalus membranaceus*–*Salvia miltiorrhiza*, *Coptis chinensis*–*Anemarrhena asphodeloides*, *Rehmannia glutinosa*–*Ophiopogon japonicus*, Trichosanthes kirilowii–Anemarrhena asphodeloides, *Panax ginseng*–*Schisandra chinensis*, *Salvia miltiorrhiza*–*Ligusticum chuanxiong*,	Liuwei Dihuang pill, Jinkui Shenqi pill, Yuye decoction, Xiaoke formula, Yunü decoction, Shengmai powder, Shenling Baizhu powder, Buzhong Yiqi decoction, Xuefu Zhuyu decoction, Taohong Siwu decoction, Qiwei Baizhu powder, Gegen Qinlian decoction,

### 3.2. Metabolomics Studies on TCM Syndromes in Diabetes

Metabolomics shares a close biological association with TCM syndromes, where syndromes represent macroscopic pathological manifestations of the body in specific spatiotemporal contexts, while metabolomics elucidates underlying microscopic metabolic dynamics from a systems biology perspective. This integration is particularly valuable for diabetes, as global metabolomics research has clearly characterized the core metabolic dysregulation of the disease, and TCM syndrome‐specific metabolic signatures can be contextualized within this broader framework to enhance international relevance.

Qi‐yin deficiency (QYD) syndrome, a common TCM syndrome characterized by fatigue, dry mouth, and reduced organ function, is the core syndrome in the mid‐stage of diabetes and the most common and prolonged clinical phase, marking the transition from metabolic hyperactivity to organ functional decline. Notably, the metabolic disturbances associated with QYD align with the well‐established metabolic phenotypes of diabetes, including altered amino acid metabolism and oxidative stress‐related lipid metabolism reported in non‐TCM biomedical studies [15]. Huang et al. [16] employed LC‐MS/MS (Thermo Q Exactive) with a C18 column (2.1 × 150 mm, 2.5 μm) and gradient elution (A: 5 mM ammonium acetate; B: methanol) to profile serum metabolites. Data preprocessed by XCMS and SIMCA revealed 7 differential metabolic pathways, with L‐glutamate and arachidonic acid identified as biomarkers (VIP > 1, *p* < 0.05) via PLS‐DA and *t*‐test. The high sensitivity of LC‐MS/MS (LOD = 0.01–0.1 ng/mL) ensured detection of low‐abundance arachidonic acid, including D‐glutamine and D‐glutamate metabolism, which is consistent with the core amino acid metabolic dysregulation in diabetes identified by Pandey et al. [17]. Among differential metabolites, L‐glutamate and arachidonic acid were identified as potential biomarkers for T2DM QYD syndrome. Arachidonic acid is a key mediator of inflammatory metabolic disturbances in diabetes and has been extensively characterized by MS‐based metabolomics. Qiu et al. [18] detected 40 differential metabolites in urine from QYD rats, with 18 downregulated and 22 upregulated, enriched in pathways involving carbohydrate, lipid, amino acid, and bioenergy metabolism—consistent with the universal metabolic disturbance patterns of diabetes summarized by Xu et al. [15]. This study further supports the alignment between TCM syndrome‐related metabolic changes and the global cognitive framework of diabetes metabolic pathology, as the identified carbohydrate and energy metabolism disturbances are core features of insulin resistance and β‐cell dysfunction. Turbid toxin accumulation (TTA) syndrome is the core syndrome in the mid‐to‐late stages of diabetes complications, a TCM syndrome associated with metabolic waste accumulation and complications like microangiopathy. Zhao et al. [19] screened 17 potential differential metabolites in plasma from T2DM patients with TTA, with seven upregulated (e.g., D‐4′‐phosphopantothenate and 2,6‐dichloroindophenol) and ten downregulated (e.g., 3‐methoxybenzoic acid and 3‐iodooctadecanoic acid). The key pathway identified was pantothenate and CoA biosynthesis, which is jointly involved by D‐4′‐phosphopantothenate and β‐alanine. Pantothenate and CoA biosynthesis dysfunction has been widely validated in global diabetes metabolomics studies as a key pathway linked to mitochondrial dysfunction, energy metabolism disturbance, and diabetic microvascular complications. It is directly associated with the classical characterization of lipid metabolic remodeling [20].

**FIGURE 3:**
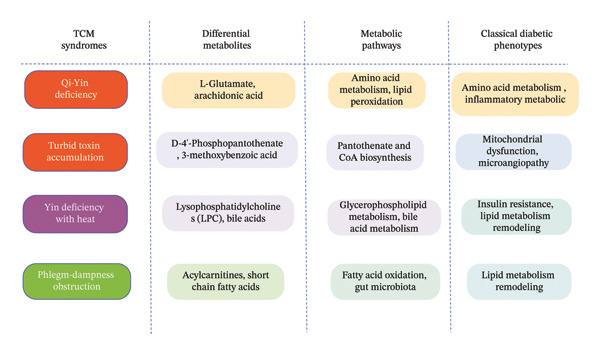
Schematic of the correlation between traditional Chinese medicine syndromes and classical diabetic metabolic phenotypes.

The syndrome of yin deficiency with excessive heat is one of the common syndromes of diabetes mellitus, which is mostly seen in the early or middle stage of the disease, and a number of metabolomic studies have been carried out on it. Through urinary metabolomics analysis, Huang Hanhui found that this syndrome type is closely associated with disorders in glycerophospholipid metabolism, bile secretion, steroid degradation, and primary bile acid biosynthesis [21]. Glycerophospholipid metabolic disturbance is a core feature of diabetes metabolic dysregulation reported in global metabolomics research, with phosphatidylcholines (PCs) and LPCs being consistently identified as differentially expressed metabolites in diabetic patients across multiethnic cohorts, and their abnormal levels are closely linked to insulin resistance and β‐cell dysfunction [22, 23]. The phlegm‐dampness obstruction syndrome is more common in the early stage of diabetes. Relevant metabolomics studies have mainly focused on diabetic patients with phlegm‐dampness constitution, and it has been found that this syndrome type is closely related to abnormalities in gut microbiota metabolites, with core metabolites involving pathways such as fatty acid oxidation and bile acid metabolism [24]. This confirms that metabolomics technology can reveal the intrinsic correlation between “endogenous phlegm‐dampness” of this syndrome type and metabolic disorders, and the disorder of fatty acid oxidation in this TCM syndrome is highly consistent with the abnormal lipid metabolism of diabetes characterized by elevated free fatty acids (FFAs) and acylcarnitines in mainstream biomedical research. In fact, acylcarnitines, as key metabolites of fatty acid β‐oxidation, have been validated as robust biomarkers for predicting the progression of diabetes and its complications such as diabetic kidney disease (DKD) in large‐scale prospective cohort studies [25]. Figure 3 illustrates the correspondence between TCM syndromes and classic metabolic phenotypes.

### 3.3. Metabolomics in Studying the Material Basis of TCM for Diabetes Treatment

TCM has a long history in diabetes treatment, with its multicomponent and multitarget properties effectively alleviating symptoms and improving metabolic disorders, offering unique advantages for complex diseases. However, insufficient research on the material basis of TCM efficacy has hindered its modernization. Metabolomics, leveraging modern analytical techniques, evaluates metabolic changes holistically and has become a vital tool for elucidating the material basis of TCM compounds, with widespread applications.

Khanna et al. [26] used UPLC‐QTOF‐MS to chemically analyze *Cupressus torulosa* needle extracts, tentatively identifying 50 components. The hypoglycemic active substances were primarily compounds such as epicatechin, amentoflavone, and cupressuflavone, along with natural iridoid glycosides exemplified by harpagoside. Li et al. [27] found that ferulic acid‐4‐β‐glucoside, bufotenine, jatrorrhizine, and berberine (BBR) from *Berberis vernae* exhibited significant affinity for 30 proteins related to T2DM and 14 differential metabolite‐associated proteins, confirming these as potential active components for T2DM. To further clarify the specific metabolic biomarkers of antidiabetic Chinese medicinal materials (single herbs, herb pairs, and classic formulas) identified by diverse metabolomic approaches, the corresponding detection techniques, biological samples, and characteristic differential biomarkers are summarized in Table 3.

**TABLE 3:** Metabolomic detection of specific biomarkers in antidiabetic Chinese herbal medicines.

Chinese herbal medicine (single/herb pair/formula)	Metabolomic technology	Biological sample	Specific biomarkers (VIP > 1, *p* < 0.05)	Reference
*Berberis vernae*	^1^H NMR	Serum	Isoleucine, trimethylamine N‐oxide, glucose	[27]
Gegen Qinlian decoction (formula)	Targeted LC‐MS/MS	Feces	Deoxycholic acid, ursodeoxycholic acid, taurodeoxycholic acid, α‐muricholic acid	[28]
*Anemarrhena asphodeloides* (single herb)	Lipidomics/LC‐MS/MS	INS‐1 cell lysate	Phosphatidylcholine, phosphatidylserine, phosphatidylethanolamine, phosphatidylglycerol	[29]
Huanglian decoction (formula)	HPLC‐MS	Urine	Cytosine, L‐carnitine, betaine, glucose	[30]
*Morus alba* (mulberry leaf, single herb)	LC‐MS/MS	Liver tissue	α‐D‐Glucose, glycerol phosphate, succinate, fumarate	[31]
Yu‐Ye Tang (formula)	LC‐MS/MS	Serum	1‐Oleoyl‐sn‐glycero‐3‐phosphocholine, 5‐hydroxytryptophan, L‐kynurenine, 1‐acyl‐sn‐glycero‐3‐phosphocholine (18:1)	[32]
*Sophora flavescens* (single herb)	LC‐MS/MS	Retinal tissue	Taurine, LPC (18:2 (9Z, 12Z)/0:0)), arginine, vitamin A2	[33]
*Phellodendri chinensis* Cortex–*Anemarrhenae rhizoma* herb pair	HPLC‐Q‐TOF/MS	Human serum	2‐Hydroxyvaleric acid, hypoxanthine, inosine, arachidonic acid	[34]

### 3.4. Metabolomics in Elucidating Mechanisms of TCM in Diabetes Treatment

TCM, as a traditional therapeutic approach, has developed advantages in regulating glucose and lipid metabolism and combating insulin resistance through sustained practice, experience, and observational treatments, offering mild yet stable long‐term effects [35–37]. Although its efficacy has been clinically validated, the active substances and modes of action have long lacked scientific elucidation. This stems from TCM compounds featuring multiple active components that achieve disease treatment and regulation via multicomponent, multitarget synergies [38]. Thus, interpreting the pathological substances and pathways in complex syndromes to comprehensively evaluate TCM formulas’ effects has become a focus in modern medicine, where metabolomics fulfills this need [39]. Metabolomics has revealed five mechanisms of TCM in diabetes treatment.

#### 3.4.1. Improving Insulin Resistance

Insulin resistance reduces physiological sensitivity to insulin, impairing glucose uptake by tissues under normal insulin concentrations, representing a core pathological basis for diabetes onset and progression. Metabolomic investigations have comprehensively delineated the core metabolic dysregulations driving insulin resistance in diabetes, with conserved perturbations in amino acid, lipid, and energy metabolism validated across independent cohorts and populations [40]. Accumulating evidence further identifies dysregulated branched‐chain amino acid (BCAA) and sphingolipid metabolism as two pivotal pathways underlying the pathogenesis of insulin resistance [41, 42]. Metabolomic studies have revealed that TCMs such as mulberry leaf and *Pueraria* root can reverse the dysregulation of amino acid and lipid metabolic pathways through their regulatory effects, thereby ameliorating insulin resistance. Ma et al. [43] used serum metabolomics to study mulberry leaf effects on T2DM rat serum metabolites, involved in amino acid, carbohydrate, and lipid metabolism, contributing to obesity and insulin resistance amelioration. Cai et al. [44] explored puerarin’s role in diabetes, with metabolomics showing regulation of sphingolipid metabolism and reduced ceramide levels, which positively correlate with insulin resistance index and T2DM risk, as key factors in insulin resistance. Zhou et al. [44] characterized metabolite profiles in high‐fat diet‐induced KK‐Ay mice using LC‐MS‐based untargeted metabolomics, finding ginsenoside Rb1 reduced fecal FFA levels, includingα‐linolenic acid, oleic acid, arachidonic acid, palmitic acid, and stearic acid. FFAs are closely linked to insulin resistance, with high concentrations disrupting insulin signaling via multiple mechanisms, and the downregulation of these FFAs by ginsenoside Rb1 mirrors the therapeutic direction of targeting lipid metabolic dysregulation in diabetes, a core pathway validated by translational metabolomic studies linking lipid signatures to clinical insulin resistance outcomes.

#### 3.4.2. Protecting Pancreaticβ‐Cell Function

Pancreaticβ‐cells are central to diabetes pathogenesis, with functional defects and reduced numbers spanning the entire disease process. Global metabolomics and clinical cohort studies have well‐characterized the core metabolic perturbations underlying pancreatic β‐cell dysfunction in diabetes, including disordered glucose metabolism, impaired tricarboxylic acid (TCA) cycle flux, and lipid metabolic remodeling with elevated ceramides and FFAs [17, 20] [1, 2]. Shi et al. [29] extracted timosaponin BII from *Anemarrhena asphodeloides* and investigated its protective effects onβ‐cells in T2DM using a glycolipid toxicity INS‐1 cell model. Lipidomics identified phosphatidylserine as a lipid marker; supplementation enhanced glucose‐stimulated insulin secretion and reducedβ‐cell apoptosis, confirming timosaponin BII’s role in restoring insulin secretion and cell viability. Zhang et al. [45] studied *Coptis deltoidea* in T2DM mouse models, with metabolomics revealing inhibition of overactivated glycolysis and regulation of TCA cycle products. Hyperactive glycolysis and impaired TCA cycles lead toβ‐cell dysfunction and apoptosis, elucidating *C. deltoidea’s* protective mechanism. This regulatory effect on central carbon metabolism is consistent with the established metabolic profile of diabetes, where disrupted glycolysis–TCA cycle coupling is a key driver of β‐cell energy metabolism disorder and functional failure [20].

#### 3.4.3. Regulating Glycolipid Metabolism

Interactions between hyperglycemia and dyslipidemia form a vicious cycle of glycolipid toxicity, synergistically driving diabetes progression and the development of macrovascular and microvascular complications, with core perturbations in glucose metabolism, lipid remodeling, and amino acid metabolism being the hallmarks of this pathological process. Numerous studies have demonstrated that the regulatory effects of TCM formulas and bioactive components on glucose and lipid metabolism are highly consistent with the core metabolic dysregulations of diabetes identified in extensive biomedical research: both target key nodes such as glucose catabolism, amino acid transamination, and lipid β‐oxidation. Pan et al. [30] analyzed rat urine post‐Huanglian decoction treatment using HPLC‐MS‐based urinary metabolomics, showing regulation of biomarkers like cytosine, L‐carnitine, phenylalanine, glucose, citric acid, and phenylpyruvic acid, linked to energy metabolism—among which L‐carnitine and phenylalanine are well‐characterized metabolites with altered levels in clinical diabetic populations, and their dysregulation is closely associated with insulin resistance and lipid metabolic disorder. Li et al. [46] focused on alkaloids, flavonoids, and polysaccharides from mulberry leaves, using urinary metabolomics to assess differential metabolites in pathways, demonstrating hypoglycemic activity via glucose, amino acid, and lipid metabolism regulation, confirming mulberry leaves’ efficacy in T2DM; the study’s findings align with global metabolomics evidence that branched‐chain amino acids (BCAAs) and FFAs are key metabolic signatures of T2DM, and the modulation of these metabolic axes is a critical therapeutic target for improving glycolipid homeostasis [20]. Song et al. [47] found Jinqi Jiangtang tablets improve T2DM primarily by regulating glucose and lipid metabolism pathways. Ma et al. [32] used untargeted metabolomics to show Yu‐Ye Tang regulates arachidonic acid metabolism, alanine, aspartate, and glutamate metabolism in T2DM rats, indicating its therapeutic role via glycolipid metabolism modulation.

#### 3.4.4. Regulating Gut Microbiota

The association between gut microbiota and diabetes is a frontier in metabolic disease research, forming a complex bidirectional regulatory network via the “microbiota‐gut‐brain/liver/pancreas axis. Environmental factors including diet, microbial composition, and host metabolic interactions are key regulators of the metabolome, and the dysregulated gut microbial profile in obesity and diabetes is closely linked to the onset and progression of metabolic disorders [17].” Liu et al. [28] used targeted metabolomics to show that Gegen Qinlian decoction with dried ginger treats T2DM mice by modulating gut microbiota and bile acid metabolism. Yao et al. [48] demonstrated *Cyclocarya paliurus* polysaccharides alleviate T2DM symptoms via gut microbiota and short‐chain fatty acid (SCFA) modulation using untargeted metabolomics. Wang et al. [49] detected metabolite changes with metabolomics, showing Danggui Buxue decoction regulates pathways like alanine, aspartate, and glutamate metabolism in gut microbiota, restoring balance in T2DM rats for antidiabetic effects—these amino acid metabolic pathways are core dysregulated pathways in diabetes, with elevated alanine, aspartate, and glutamate being consistent with the plasma metabolomic signatures of insulin resistance and T2DM reported in large‐scale human cohort studies [50]. Ma et al. [51] found PuRenDan modulates gut microbiota metabolites like pantothenic acid, 1‐methylhistamine, and 1‐methylhistidine, involved in pantothenate and CoA biosynthesis, histidine metabolism, and secondary bile acid biosynthesis. Correlation analyses revealed close associations between gut microbiota, metabolites, and T2DM indices. Analyses of these studies on TCM have revealed intimate correlations between the gut microbiota, metabolites, and T2DM‐related indices, which align with the global consensus that host metabolic health can be modulated by reshaping the metabolome.

#### 3.4.5. Improving Microcirculation to Prevent Complications

**FIGURE 4:**
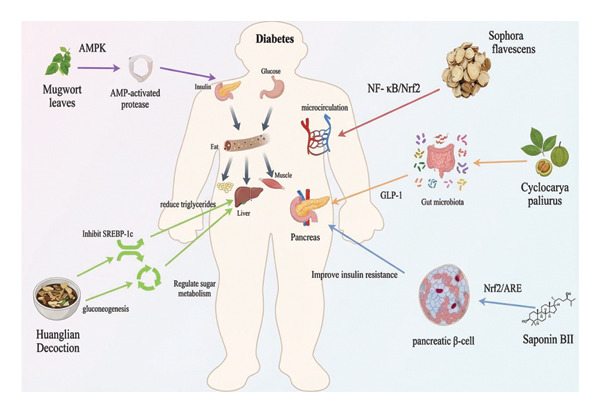
Mechanisms of traditional Chinese medicine in treating diabetes.

Diabetic microangiopathy, a hallmark chronic complication, involves systemic damage to microvessels under prolonged hyperglycemia, primarily affecting the retina, kidneys, nerves, and skin microcirculation, potentially leading to organ failure. It is important to clarify that diabetic microangiopathy is driven by the interplay of metabolic disorders and chronic low‐grade inflammation. The combined effects of these two factors, induced by long‐term hyperglycemia, lead to endothelial damage and microcirculatory dysfunction, ultimately resulting in diabetic complications. Metabolomic studies have clearly demonstrated that diabetic microangiopathy is closely associated with core metabolic dysregulations, including glycerophospholipid remodeling, amino acid metabolic disorders, mitochondrial energy metabolism dysfunction, and oxidative stress‐induced imbalances in lipid and fatty acid metabolism in diabetes [52]. Additionally, multiple studies have shown that TCM can treat related complications by improving microcirculation. Luo et al. [33] studied *Sophora flavescens* extract’s material basis for diabetic retinopathy, screening 39 differential metabolites via metabolomics, with 23 influenced by the extract. It acts via pathways like lactose metabolism, bile acid metabolism, and glycerophospholipid metabolism, delaying retinopathy progression through oxidative stress, inflammation, and vascular dysfunction linkages. Xu et al. [53] used metabolomics to study salvianolic acid B and tanshinone IIA from *Salvia miltiorrhiza*, regulating saturated fatty acid metabolism, glycerophospholipid metabolism, and steroid biosynthesis, modulating glomerular, vascular, and mesangial functions via oxidative stress, inflammation, and fibrosis to improve early diabetic nephropathy in rats. Zhao et al. [54] found Jinmaitong ameliorates diabetic peripheral neuropathy by regulating abnormal energy balance and mitochondrial function, improving peripheral nerve function and symptoms. The mechanisms underlying the therapeutic effects of *Sophora flavescens*, *Salvia miltiorrhiza*, and Jinmaitong on diabetes are consistent with the findings of global metabolomic studies [20]. In recent years, metabolomics has revealed mechanisms of various TCMs for T2DM. Details are shown in Table 4. Figure 4 illustrates the mechanisms of TCM in diabetes treatment. The abbreviations used in Figure 4 are defined as follows: nuclear factor‐kappa B (NF‐κB), adenosine 5′‐monophosphate‐activated protein kinase (AMPK), nuclear factor e2‐related factor 2 (Nrf2), antioxidant response element (ARE), and glucagon‐like peptide‐1 (GLP‐1).

**TABLE 4:** Examples of metabolomics applications in TCM treatment of diabetes.

Drug name	T2DM model	Method	Sample detected	Main marker observed	Sample size (metabolomics analysis)	Biological matrix consistency	Mechanism of action	Author
Wendan decoction	Phlegm‐dampness obstructive sleep apnea‐hypopnea syndrome with T2DM patients	Untargeted metabolomics	Serum	DL‐Arginine, guaiacol sulfate, azelaic acid, phloroglucinol, uracil	*n* = 20 patients	Consistent (serum)	Regulates biomarkers like DL‐arginine, guaiacol sulfate, azelaic acid, phloroglucinol, and uracil; significantly improves glycolipid metabolism and insulin resistance	Zhou [55]
*Lomatogonium rotatum*	High‐fat diet combined with STZ‐induced mice	Untargeted metabolomics	Serum	Vitamin B6, mevalonate‐5P, D‐proline, L‐lysine, taurine	10 mice/group	Consistent (serum)	Regulates insulin resistance by altering levels of vitamin B6, mevalonate‐5P, D‐proline, L‐lysine, and taurine	Dai [56]
Pi‐Dan‐Jian‐Qing decoction	High‐fat diet combined with STZ‐induced mice	Untargeted metabolomics	Serum and feces	DL‐Tryptophan, L‐kynurenine, D‐(+)‐malic acid, citric acid	6 rats/group	Consistent (serum)	Improves gut microbiota and insulin resistance by increasing relative abundance of *Lactobacillus*, *Blautia*, *Bacteroides,* etc., and regulating tryptophan metabolism, histidine metabolism, etc.	Xie [57]
*Cynomorium songaricum* polysaccharide	High‐fat diet‐induced mice	Targeted metabolomics	Serum	Lysophosphatidylcholine (LPC) 14:0, sphingomyelin (SM) d16:1/24:1, lysophosphatidylethanolamine (LPE) 22:0, phosphatidylcholine (PC) 16:1/18:2	8 mice/group	Consistent (serum)	Improves lipid metabolism by regulating phosphatidylcholine, lysophosphatidylcholine, phosphatidylethanolamine, and sphingomyelin metabolites	Shi [58]
*Phellodendri chinensis* Cortex–*Anemarrhenae rhizoma* herb pair	T2DM patients, high‐fat diet combined with STZ‐induced mice	Untargeted metabolomics	Serum	Hypoxanthine, inosine, 2‐hydroxyvaleric acid, docosahexaenoic acid	Patients: 250; mice: 10/group	Consistent (serum)	Improves lipid metabolism and insulin resistance by regulating unsaturated fatty acid biosynthesis and purine metabolism	Yu [34]
*Schisandra chinensis* polysaccharide	High‐fat diet combined with STZ‐induced mice	Untargeted metabolomics	Serum and feces	Vitamin A, all‐trans‐4‐hydroxyretinoic acid, retinoyl b‐glucuronide, dehydroepiandrosterone	6 mice/group	Consistent (serum)	Improves gut microbiota composition and short‐chain fatty acid levels	Guo [59]
*Rubus irritans*	High‐fat diet combined with STZ‐induced mice	Untargeted metabolomics	Serum	Inosine, glycolate, L‐threonate, β‐glycerophosphate	6 rats/group	Consistent (serum)	Improves glycolipid metabolism and insulin resistance by regulating ascorbate and aldarate metabolism, ABC transporters, glycerophospholipid metabolism, and carbon cycle pathways	Han [60]
Red ginseng	High‐fat diet combined with STZ‐induced mice	Untargeted metabolomics	Serum	D‐Ornithine, taurodeoxycholic acid, lysophosphatidylcholine (24:1 (15Z)), 2‐methylbutyroylcarnitine	6 rats/group	Consistent (serum)	Improves lipid metabolism and insulin resistance by regulating D‐arginine and D‐ornithine metabolism, D‐glutamine and D‐glutamate metabolism, etc.	Yang [61]
Yuquan pill	High‐fat diet combined with STZ‐induced mice	Untargeted metabolomics	Serum	Lenticin, 1,2,3‐trihydroxybenzene, sorbitol, methyl (indol‐3‐yl) acetate	8 rats/group	Consistent (serum)	Improves insulin resistance and gut microbiota by regulating five pathways like ascorbate and aldarate metabolism, nicotinate and nicotinamide metabolism, and abundance of six genera including Firmicutes and Bacteroidetes	Liu [62]
White hyacinth bean	High‐fat high‐sugar diet with low‐dose STZ‐induced mice	Targeted metabolomics	Serum	L‐Valine, isoleucine, L‐arginine, LPC (P‐18:1 (9Z))	8 rats/group	Consistent (serum)	Improves insulin resistance and protects β‐cells by regulating four pathways like phenylalanine metabolism and tryptophan metabolism	Wang [63]
*Cornus officinalis*	High‐fat diet combined with STZ‐induced mice	Untargeted metabolomics	Serum and urine	Taurine, chenodeoxycholic acid, glycocholic acid, L‐tyrosine	9 rats/group	Inconsistent (serum + urine)	Improves glycolipid metabolism and insulin resistance by regulating 42 metabolites like taurine, glycochenodeoxycholic acid, glucuronic acid, and L‐tyrosine	Hou [64]
Da‐Chai‐Hu decoction	High‐fat diet combined with STZ‐induced mice	Untargeted metabolomics	Plasma	Hydroxyphenylacetylglycine, phenylglyoxylic acid, hippuric acid, p‐cresol sulfate	10 rats/group	Consistent (urine)	Improves insulin resistance and protects 尾‐cells via AGEs/RAGE/AKT signaling pathway	Wang [65]
Huidouba	High‐fat diet combined with STZ‐induced mice	Untargeted metabolomics	Plasma	L‐Valine, betaine, N6‐acetyl‐L‐lysine, creatine	13 rats/group	Consistent (plasma)	Improves glucose metabolism and microangiopathy by regulating bile acid metabolism	Li [66]
*Salvia miltiorrhiza*	db/db mice	Untargeted metabolomics	Urine	Niacinamide, homovanillin, tyramine, dopamine	6 mice/group	Consistent (urine)	Improves insulin resistance via PTP1B/IRS‐1/AKT signaling pathway	Zhang [67]
Shouhui Tongbian capsule	db/db mice and high‐fat diet‐fed mice	Targeted metabolomics	Feces and plasma	Isobutyric acid, isovaleric acid, 2‐methylbutyric acid, acetic acid	8‐10 mice/group	Inconsistent (feces + plasma)	Improves gut microbiota and insulin resistance by regulating gut BCAAs‐mTORC1/IRS‐1/PI3K/AKT axis	Wang [68]
Mulberry	High‐fat high‐sucrose diet with low‐dose STZ‐induced mice	Untargeted metabolomics	Feces and plasma	Glycine, Phe‐Pro, urocanic acid, phylloquinone	6 mice/group	Consistent (feces)	Improves gut microbiota by regulating abundance of four genera like *Prevotella* and *Parabacteroides*	Du [69]
*Cordyceps militaris* extract	High‐fat diet combined with STZ‐induced mice	Untargeted metabolomics	Feces	Catechin, erucic acid, phenols, pyrimidine nucleosides	10 mice/group	Consistent (feces)	Regulates gut microbiota by increasing abundance of Firmicutes and Bacteroidetes	Liu [70]
Xiexin decoction	High‐fat diet combined with STZ‐induced mice	Untargeted metabolomics	Feces	Valine, aspartic acid, phosphoenolpyruvic acid, succinic acid	6 rats/group	Consistent (feces)	Improves gut microbiota and insulin resistance and protects 尾‐cells by regulating arachidonic acid metabolism, amino acid metabolism, and bile acid metabolism	Qian [71]
*Malus toringoides*	db/db mice	Untargeted metabolomics	Feces	Acetic acid, butyric acid, malonic acid, glutaric acid	6 mice/group	Consistent (feces)	Improves gut microbiota and glycolipid metabolism by regulating abundance of four genera like *Alloprevotella* and *Parabacteroides*	Zheng [72]
Renshenjian decoction	High‐fat diet combined with STZ‐induced mice	Untargeted metabolomics	Liver tissue	Ceramide, ornithine, leucine, LPC (16:0)	8 mice/group	Consistent (liver tissue)	Improves insulin resistance by regulating HIF‐1伪/SPTLC2 pathway	Chen [73]
Zuogui Jiangtang Qinggan formula	db/db mice	Targeted metabolomics	Liver tissue	Triglyceride (TG), diglyceride (DG), phosphatidylcholine (PC), phosphatidylethanolamine (PE)	6 mice/group	Consistent (liver tissue)	Improves lipid metabolism by regulating levels of triglycerides, diglycerides, and phosphatidylcholine	Zhou [74]
Mulberry leaf	High‐fat high‐sugar diet with STZ‐induced mice	Untargeted metabolomics	Liver tissue	α‐D‐Glucose, glycerol phosphate, succinate, fumarate	9 rats/group	Consistent (liver tissue)	Improves glycolipid metabolism and insulin resistance by regulating key enzymes like ACSL5, Dlat, Pdhb, G6pc, Mdh2, and Cs	Lv [31]
Buyang Huanwu decoction	High‐fat diet‐induced mice	Targeted metabolomics	Liver tissue	Triglyceride (TG) (18:4_18:2_18:3), phosphatidylcholine (PC) (36:2e), diglyceride (DG) (22:6_22:6), sphingomyelin (SM) (d18:1_24:0)	8–10 rats/group	Consistent (liver tissue)	Improves gut microbiota and lipid metabolism by regulating abundance of Bacteroidetes and Firmicutes	Liu [75]
*Dendrobium officinale*	db/db mice	Untargeted metabolomics	Liver tissue	Acetic acid, propanoic acid, butyric acid, PPAR pathway‐related metabolites	6 mice/group	Inconsistent (liver tissue, faeces)	Improves gut microbiota and insulin resistance by regulating short‐chain fatty acids and PPAR pathway	Song [76]
Shenqi compound	GK mice	Untargeted metabolomics	Intestinal contents	L‐Tyrosine, isoleucyl‐tyrosine, leucyl‐hydroxyproline, cyclohexanecarboxylic acid	10 rats/group	Consistent (intestinal contents)	Improves gut microbiota and glycolipid metabolism by regulating seven genera like *Prevotella* and *Lactobacillus* and five metabolisms like gluconeogenesis/glycolysis, amino acid metabolism, and lipid metabolism	Zhang [77]
Shenshu Tiaopi granule	GK rats	Untargeted metabolomics	Intestinal contents	2‐Deoxyglucose, D‐maltose, alpha‐lactose, beta‐muricholic acid	8 rats/group	Consistent (ileal contents)	Improves gut microbiota and lipid metabolism by regulating abundance of *Monoglobus* and bile acid biosynthesis and cholesterol metabolism	Zhao [78]
*Angelica* polysaccharide	High‐fat high‐sugar diet‐induced mice	Untargeted metabolomics	Intestinal contents	L‐Cysteine, glycerophospholipids, N‐methyltryptamine, D‐sedoheptulose 7‐phosphate	5 mice/group	Consistent (intestinal contents)	Improves gut microbiota by regulating genera like *Akkermansia* and *Lactobacillus*	Tang [79]
Erzhi pill	High‐fat diet with low‐dose STZ‐induced mice	Untargeted metabolomics	Heart	Glutamine, taurine, glycine, 3‐hydroxybutyrate (3‐HB)	5 rats/group	Consistent (heart tissue)	Improves glucose metabolism, insulin resistance, and microangiopathy by regulating AMPK and PI3K/Akt/FoxO3a signaling pathways	Peng [80]

### 3.5. Metabolomics in Evaluating TCM Efficacy for Diabetes Treatment

Traditional TCM efficacy assessments primarily rely on subjective symptom scores and routine biochemical indicators (e.g., blood glucose and serum insulin). While valuable, these are limited by subjectivity, individual variability in symptom perception, and lagged, simplistic evaluation criteria. Metabolomics, through objective dynamic metabolite changes, reveals holistic effects of herbal interventions, providing modern scientific support for optimizing classic formulas and personalized medication. Global metabolomics studies have well‐established that T2DM is characterized by core dysregulations in amino acid metabolism (especially BCAAs), lipid remodeling, energy metabolism, and inflammatory metabolic disturbances [81]. Currently, a large‐scale population cohort study has confirmed the associations between amino acid metabolic dysregulation, lipid disorders, and the risk of T2DM onset and clearly identified metabolites as robust biomarkers for disease progression and therapeutic response [82]. These universal metabolic signatures provide an essential reference framework for objectively evaluating the efficacy of TCM interventions. Based on the above elucidated antidiabetic mechanisms of TCM, the therapeutic efficacy of potent TCM interventions (including bioactive extracts, classic formulas, and optimized formulations) and their core metabolic modulation effects identified by metabolomics are summarized in Table 5.

**TABLE 5:** Therapeutic application of potent antidiabetic Chinese herbal medicines elucidated by metabolomics.

Potent TCM interventions	Diabetic model/subjects	Metabolomic approach	Key therapeutic efficacy	Core metabolic modulation	Clinical application prospect	Reference
Berberine‐baicalin (10∶1)	T2DM patients, damp‐heat internal accumulation type T2DM fecal microbiota transplantation pseudo‐sterile mice	Untargeted UPLC‐QTOF‐MS	Improve insulin resistance, regulate gut microbiota structure	Mainly modulate arachidonic acid metabolism, α‐linolenic acid metabolism	Personalized hypoglycemic therapy	[83]
*Pueraria lobata* polysaccharides	C57BL/KsJ‐db/db mice + high‐fat‐diet + STZ‐induced T2DM mice	UPLC‐QTOF‐MS	Improve insulin resistance, regulate lipid metabolism	PPAR pathway, sphingolipid metabolism	Novel strategy for diabetic hepatopathy	[44, 84]
Jinqi Jiangtang tablets	HFD + STZ mice	UPLC‐QTOF‐MS	Regulate glycolipid metabolism, reduce hyperglycemia	Target glucose and lipid metabolic pathways	Clinical oral preparation for T2DM	[47]
*Posidonia oceanica* extract (nano‐formulation)	STZ mice	Untargeted GC‐MS	Enhance hypoglycemic effect, improve metabolic homeostasis	Downregulate glucose/leucine levels, modulate lipid metabolism	Formulation optimization for TCM bioactive compounds	[85]
*Salvia miltiorrhiza* extract	db/db mice	UPLC‐TOF‐MS	Improve glucose/lipid metabolism; alleviate liver/kidney damage	Regulate glycerophospholipid metabolism, inhibit oxidative stress	Potential therapy for early diabetic nephropathy	[53]

Chen et al. [83] used UPLC‐Q‐TOF‐MS to study BBR and baicalin (BAI) combinations in improving T2DM, identifying 28 differential metabolites and 14 pathways consistent with gut microbiota findings, multidimensionally indicating that Coptis–Scutellaria compatibility protects against T2DM by modulating gut microbiota and fecal metabolites. This aligns with global metabolomic evidence that gut microbiota–host co‐metabolism is a key node in T2DM pathogenesis, and restoring microbial–metabolic homeostasis is a validated strategy for improving insulin resistance and metabolic disorders [17, 85]. Ammar et al. compared antidiabetic potential of *Posidonia oceanica* extract and its gelatin nanoparticle formulation using untargeted GC‐MS metabolomics, showing the nanoparticle formulation as more effective. Such comparative metabolomic evaluation echoes translational metabolomics principles that metabolic profile shifts can sensitively reflect therapeutic potency and formulation optimization, which is increasingly adopted in international antidiabetic drug development [20]. Zhao et al. [86] evaluated lifestyle and TCM interventions on prediabetes indicators, identifying N‐neuraminylmethionine and 5‐hydroxy‐L‐tryptophan as core potential biomarkers for Xiaokeqing granule and lifestyle efficacy. This practice of defining efficacy‐related metabolites is consistent with international standards for diabetes metabolomics, where metabolite panels are validated across cohorts to predict therapeutic responses and stratify high‐risk individuals [87]. Zhang et al. [88] assessed exercise therapy’s potential impact on T2DM via untargeted metabolomics of serum and urine from healthy, Tai Chi, and brisk walking groups, revealing impaired carbohydrate, lipid, and amino acid metabolism in T2DM, improved by exercise. Tai Chi ameliorates disrupted BCAA metabolism, while brisk walking regulates steroid hormone biosynthesis and arachidonic acid metabolism. BCAA elevation is a well‐documented metabolic hallmark of insulin resistance and T2DM risk in large‐scale prospective cohorts worldwide [20], so the regulation of BCAAs by Tai Chi directly targets a core pathogenic metabolic abnormality, providing high‐level mechanistic evidence for its antidiabetic efficacy. According to the Chinese Pharmacopoeia, *Pueraria lobata* and *Pueraria thomsonii* treat xiao ke to lower blood glucose; Wang et al. [84] compared *Pueraria lobata* and *Pueraria thomsonii* polysaccharides via gut microbiota and metabolomics, showing no significant efficacy differences, both treating T2DM by modulating peroxisome proliferator‐activated receptors (PPAR) signaling to regulate insulin resistance. PPAR signaling is a central pathway governing lipid and glucose homeostasis, and its metabolic consequences (e.g., fatty acid oxidation and glucose uptake) are consistently mapped in global diabetes metabolomics as key pathways for therapeutic intervention [89]. The summary of metabolomics studies on the treatment of diabetes mellitus with TCM is presented in Table 6.

**TABLE 6:** Summary table of metabolomics studies on metabolic regulation and efficacy evaluation of traditional Chinese medicine and related interventions in diabetes mellitus.

Researchers (year)	Research method (metabolomics technology)	Intervention/study subjects	Observed biomarkers/differential metabolites	Associated metabolic pathways/mechanisms
Chen et al. [83]	UPLC‐Q‐TOF‐MS	Combination of berberine (BBR) and baicalin (BAI) for intervention in Type 2 diabetes mellitus (T2DM)	Alpha‐linolenic acid, 15‐keto‐prostaglandin E2, coprocholic acid, PE [16:0/20:4 (8Z, 11Z, 14Z, 17Z)]	14 differential metabolic pathways including arachidonic acid metabolism and alpha‐linolenic acid metabolism; gut microbiota–host co‐metabolism
Ammar et al. [85]	Untargeted GC‐MS metabolomics	Comparative antidiabetic potential of *Posidonia oceanica* extract and its gelatin nanoparticle formulation	Glucose, leucine, oleic acid, butane‐2,3‐diol	Amino acid metabolism pathway and lipid metabolism pathway are highly correlated with its therapeutic effects
Zhao et al. [86]	Not specified; focusing on efficacy‐related biomarker screening	Lifestyle intervention + Xiaokeqing granules for intervention in prediabetes indicators	N‐Nervonoyl methionine, 5‐hydroxy‐L‐tryptophan (core potential efficacy biomarkers)	Tryptophan metabolism pathway and pantothenate and CoA biosynthesis pathway provide a basis for efficacy prediction and high‐risk population stratification
Zhang et al. [88]	Untargeted metabolomics (serum + urine samples)	Comparison of healthy population, Tai Chi group, and brisk walking group to evaluate the effects of exercise therapy on T2DM	Leucine, isoleucine, taurine, 9‐oxo‐octadecenoic acid	Branched‐chain amino acid metabolism pathway, steroid hormone biosynthesis pathway, and arachidonic acid metabolism pathway; Tai Chi directly targets the core metabolic abnormality associated with insulin resistance (elevated BCAAs)
Wang et al. [84]	Metabolomics + gut microbiota analysis	Comparison of *Pueraria lobata* and *Pueraria thomsonii* polysaccharides for intervention in T2DM (both recorded in Chinese Pharmacopoeia for “alleviating xiao ke and lowering blood glucose”)	Alpha‐linolenic acid, tauroursodeoxycholic acid, uric acid, lysophosphatidylcholine	PPAR signaling pathway (regulates insulin resistance and participates in lipid and glucose homeostasis)

In summary, metabolomics development offers a novel perspective for TCM diabetes research. It precisely identifies differential metabolites and change patterns across syndromes, enhancing the scientificity and objectivity of TCM syndrome diagnosis and reducing the subjective bias of traditional diagnostics [90, 91]. Metabolomics’ vast data systematically depict dynamic metabolic network changes pre‐ and post‐TCM intervention, enabling broad applications in clarifying material basis, mechanisms, and efficacy evaluation of TCM compounds.

## 4. Summary and Prospects

Diabetes has become a major global health issue, yet Chinese herbal medicine boasts millennia of clinical experience. Due to the complexity of components and mechanisms, deciphering the scientific basis and systemic features of TCM in diabetes treatment remains challenging. Current research indicates metabolomics can elucidate TCM’s material basis and mechanisms for diabetes efficacy, aiding in identifying diagnostic TCM syndromes, advancing compound improvements, and developing novel antidiabetic TCM therapies. Metabolomics identifies metabolic pathways and biomarkers in forms understandable and acceptable to the international scientific community, greatly facilitating publication and exchange of TCM research in high‐impact journals and enhancing its global recognition.

The application of metabolomics in TCM for diabetes treatment faces several challenges. TCM’s reliance on subjective syndrome differentiation, without objective quantitative indicators, complicates the standardization of diagnoses and confounds metabolic markers. The multicomponent nature of TCM further complicates attributing metabolic changes to specific compounds. Additionally, metabolomics faces limitations such as the masking of low‐abundance metabolites by high‐abundance signals and the need for high‐sensitivity platforms. Small sample sizes and complex data analysis also hinder progress. To address these issues, there is a need for a unified research workflow, standardized criteria, and multicenter validation. Interdisciplinary collaboration among TCM, metabolomics, clinical medicine, and bioinformatics should be strengthened to integrate metabolic mechanisms with TCM’s holistic and syndrome differentiation principles. Focusing on internationally recognized metabolic pathways and biomarkers, a system of syndrome‐ and efficacy‐specific metabolic biomarkers should be developed.

Moreover, metabolomics‐guided biomarkers could significantly enhance personalized diabetes management, not just within the TCM framework but also in broader, globally recognized clinical settings. By identifying individual metabolic profiles and responses to therapy, these biomarkers can support the customization of treatment plans, ensuring more effective and targeted interventions. High‐sensitivity and high‐specificity detection technologies should be developed, and a comprehensive database of TCM bioactive components and their metabolic regulatory effects should be established to ensure accurate traceability of metabolic changes induced by TCM.

In conclusion, metabolomics offers a novel approach to understanding TCM’s antidiabetic mechanisms and facilitates personalized treatment. With continued technological advancement and interdisciplinary collaboration, metabolomics will play a central role in advancing both TCM‐based and global diabetes therapy.

## Author Contributions

All the authors contributed equally to each subtopic, reviewing, and final editing of the manuscript.

## Funding

This study was funded by the National Key Research and Development Program of China, 2020YFE0201800, and the Henan Province Science and Technology Research and Development Program Joint Fund (Advantageous Discipline Cultivation Category) Project, 242301420107.

## Disclosure

All contents were carefully reviewed and verified by the authors.

## Conflicts of Interest

The authors declare no conflicts of interest.

## Data Availability

The data that support the findings of this study are openly available in PubMed at https://pubmed.ncbi.nlm.nih.gov/.
